# Bovine Lactoferrin Enhances TLR7-Mediated Responses in Plasmacytoid Dendritic Cells in Elderly Women: Results From a Nutritional Intervention Study With Bovine Lactoferrin, GOS and Vitamin D

**DOI:** 10.3389/fimmu.2018.02677

**Published:** 2018-11-20

**Authors:** Marloes van Splunter, Olaf Perdijk, Henriëtte Fick-Brinkhof, Anouk L. Feitsma, Esther G. Floris-Vollenbroek, Ben Meijer, Sylvia Brugman, Huub F. J. Savelkoul, Els van Hoffen, R. J. Joost van Neerven

**Affiliations:** ^1^Cell Biology and Immunology, Wageningen University, Wageningen, Netherlands; ^2^Human Nutrition, Wageningen University, Wageningen, Netherlands; ^3^FrieslandCampina, Amersfoort, Netherlands; ^4^NIZO Food Research, Ede, Netherlands

**Keywords:** aging, TLR stimulation, pDCs, mDCs, bovine lactoferrin, GOS, vitamin D, inflammation

## Abstract

During aging the immune system is dysregulated. Especially plasmacytoid dendritic cells (pDCs) and myeloid DCs (mDCs) have reduced Toll like receptor (TLR)-mediated responses resulting in increased susceptibility to infections. Consumption of bovine lactoferrin (bLF) has been shown to reduce infections with viruses. Galacto-oligosacharides (GOS) and vitamin D are associated with reduced pro-inflammatory cytokine levels in serum, and increased TLR7/8 responses, respectively. A double-blind placebo-controlled nutritional intervention study in elderly women was performed, to investigate the potential of bLF, GOS, and vitamin D to restore TLR responsiveness of pDCs and mDCs and to reduce inflammatory markers in serum. The nutritional intervention group (*n* = 15) received bLF for 3 weeks, followed by 3 weeks of bLF + GOS, and subsequently 3 weeks of bLF + GOS + vitamin D. The placebo group (*n* = 15) received maltodextrin for 9 weeks. Every 3 weeks, blood was collected and TLR responses of pDCs and mDCs, and inflammation-related markers in serum were measured. After 3 weeks of bLF supplementation, increased TLR7/8 and TLR1/2 responses were observed in pDCs of the nutritional intervention group compared to the placebo group. When the effects of the entire nutritional intervention were investigated, increased TLR1/2 mediated responses in mDCs were observed, and in serum sVCAM tended to decrease. Finally, based on the RAND-36 questionnaire physical function tended to improve in the intervention group. Since especially TLR7-mediated responses in pDCs were enhanced after bLF supplementation compared to placebo, this suggests that bLF may contribute to antiviral responses mediated by pDC in elderly women.Clinical trial registry number: NCT03026244, clinicaltrials.gov:

## Introduction

During aging, the immune system becomes dysregulated, as indicated by two phenomena: immunosenescence and inflammaging. In immunosenescence, both the innate and adaptive immune system are dysregulated. Dysregulation of the immune system seems to involve, among others, changes in the number and function of lymphocytes and innate immune cells, as well as altered expression of Toll-like receptors (TLRs) (1–3). Because of these compromised innate and adaptive immune responses, elderly people have a decreased ability to respond to infection and vaccination (2–5). Furthermore, many age-related health disorders, such as osteoarthritis, metabolic diseases, cognitive decline, onset of frailty, and cardiovascular diseases are associated with inflammation, often referred to as inflammaging (4, 6–9). Inflammaging is associated with increased serum concentrations of pro-inflammatory cytokines (3), acute-phase proteins and soluble adhesion markers (10). The age-related reduced response to TLR stimulation is best described for myeloid dendritic cells (mDCs) and plasmacytoid dendritic cells (pDCs) (8, 11–14). Interestingly, in relation to anti-viral immune responses, pDCs of elderly people have been shown to produce lower concentrations of antiviral IFN-α and pro-inflammatory cytokines upon TLR7 and TLR9 stimulation, resulting in lowered antiviral immunity (15, 16).

Elderly people are more susceptible to severe influenza and respiratory syncytial virus (RSV) infection leading more often to hospitalization compared to adults (17) and are less responsive to influenza vaccination (8). Influenza and RSV are both single stranded RNA viruses, and the innate immune response to such viruses is mainly TLR7 mediated (14, 18). Bovine Lactoferrin (bLF) is linked to reduced number of infections by rhinoviruses and hepatitis C which are also recognized by TLR7 (19–21). bLF is an antimicrobial protein that is known to prevent sepsis, fungal infections, and enterocolitis in premature infants (22–24). In addition, bLF has been described to have anti-inflammatory effects (25, 26). Therefore, bLF might be a nutrient from milk that is able to restore TLR7 responses of pDC to viruses. Next to this, elderly have reduced serum concentrations of vitamin D in winter, which has been shown to correlate with reduced expression and responsiveness of TLR7 and TLR8 on monocytes (27). This suggests that vitamin D may impact TLR7/8 responsiveness.

In addition to direct effects on immune function, aging is also associated with microbiota changes in the gastrointestinal tract. Prebiotic oligosaccharides, such as galacto-oligosaccharides (GOS), have been shown to increase the concentrations of beneficial *Bifidobacteria* in the gut of elderly in several studies (28–30). Interestingly, consumption of GOS also reduced the concentrations of circulating pro-inflammatory cytokines (29).

The study was set up as a double-blind placebo-controlled nutritional intervention study, to investigate the potential of bLF, GOS, and vitamin D supplementation to restore TLR responsiveness of pDCs and mDCs and to reduce inflammatory cytokines in serum.

## Experimental methods

### Study set-up

The effect of bLF in combination with galacto-oligosacharides (GOS) and vitamin D in elderly women (65–85 years) was studied in a double-blind placebo-controlled nutritional intervention study. The protocol was approved by the Medical Ethics Committee of Wageningen University, the Netherlands (protocol no. NL57345.081.16), and registered at clinicaltrials.gov (identifier NCT03026244).

As TLR7 expression is X-linked (31), the study was performed in women only. Female subjects (65–85 years) were recruited. After providing informed consent, subjects were screened and were included when they were generally healthy, having a BMI 20–30, good mental status, and non-smoking. Subjects with chronic inflammatory, autoimmune, or gastrointestinal diseases or immune-compromised individuals were excluded from participation. Subjects using hormone replacement therapy, anti-inflammatory drugs (>1 × week) or immunosuppressive drugs were excluded. Furthermore, subjects were not allowed to use light therapy or go on holiday to a sunny destination. An overview of subject characteristics of the two study groups is given in Table 1. Because of the seasonal effect on vitamin D status, the study was executed in the winter period (January until March 2017).

**Table 1:** Characteristics of study participants.

**Treatment**	**Age (years) median (range)**	**BMI median (range)**	**Osteoarthritis Y/N**	**Anti-inflammatory medicine Y/N**	**Vitamine D supplementation before study Y/N**	**Blood pressure or cholesterol medication Y/N**
Intervention (*n* = 15)	74 (70–84)	23.2 (20.3–29.0)	3/12	1/14	6/9	6/9
Placebo (*n* = 15)	76 (69–85)	24.5 (20.8–29.4)	3/12	0/15	7/8	6/9

### Study design

**Figure 1:**
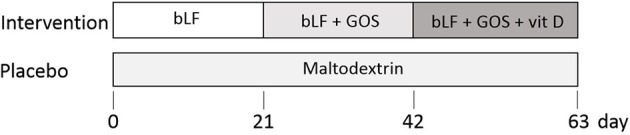
Nutritional intervention study design. The intervention group (*n* = 15) received bovine lactoferrin (bLF) for 3 weeks (days 0–21), followed by 3 weeks of bLF + Galacto-oligosacharides (GOS) (days 21–42) and 3 weeks of bLF + GOS + vitamin D. The placebo group (*n* = 15) received maltodextrin for 9 weeks (days 0–63). At study days 0, 21, 42, and 63, blood was collected.

Stratification and randomization was performed by a non-blinded person not involved in the study, all investigators were blinded until all data were collected. Women were stratified according to age, BMI, reported arthrosis, use of vitamin D supplements preceding the study, and use of medication for blood pressure or cholesterol. Subjects were randomly assigned to treatment or placebo using a random number generator. Women (*n* = 15) in the nutritional intervention group were supplemented with only bLF for 3 weeks, followed by 3 weeks bLF + GOS, followed by 3 weeks bLF + GOS + vitamin D, see Figure 1. In parallel, the placebo group (*n* = 15) received maltodextrin as placebo for bLF and GOS, and capsules filled with maltodextrin as placebo for vitamin D. Subjects were instructed to maintain their habitual diet, but to stop any vitamin D or prebiotic supplementation during the study, starting from 2 weeks before study start. Sample collection was done every 3 weeks at Wageningen University at the clinical studies facility. The primary outcome of this study was IFN-α and IL-6 production by PBMCs upon *ex vivo* TLR7 stimulation. Secondary outcomes were TNF-α production by PBMCs upon *ex vivo* TLR7 stimulation, and the percentage of IFN-α, IL-6, or TNF-α-producing pDCs in PBMCs upon *ex vivo* TLR7 stimulation.

### Sample size calculation

Since information on the impact of diet on innate immune responses in the elderly is lacking, several assumptions were made based on studies using similar outcomes as anticipated for the NOBLE study. Furthermore, it was assumed that a potential effect may be detected at any time point after intervention. Therefore, dependent and independent *t*-tests for 2 means were used as basis for sample size calculation, rather than ANOVA. Selected primary outcome of this study was IFN-α and IL-6 production by PBMCs upon TLR7 stimulation. Therefore, for sample size calculation, we used a study in which the percentage of *ex vivo* IFN-α producing pDCs was measured after TLR7 stimulation upon hormonal replacement therapy (12). In this study, the effect was an increase of 11.2% of IFN-α producing pDCs, with a pooled SD of 9.9 and an assumed rho for paired samples 0.3. Based on two-sided statistical testing (dependent samples, *t*-test), α = 0.05 (chance of type-I error) and β = 0.20 (chance of type-II error), it was calculated using the software package Statistica (2013) that at least 12 subjects per group were needed for detection of this effect at any time point after supplementation. Assuming that the placebo group would not show a change in the % IFN-α producing pDCs, 14 subjects would be needed to confirm this by comparing the intervention and placebo group with an independent *t*-test. Because of potential dropouts during the study, 1 extra subject per group was included, resulting in a group size of 15 subjects, and a total study size of 30 subjects.

### Study products

In the intervention group, subjects received 3 weeks of bLF (1.026 g/day Vivinal Lactoferrin powder, containing an active dose of bLF of 1 g/day; FrieslandCampina Domo, Amersfoort, The Netherlands). This was followed by 3 weeks of supplementation with bLF + GOS (1 g/day bLF as above; 3.67 g/day Vivinal GOS powder, containing an active dose of GOS of 2.64 g/day; FrieslandCampina Domo, Amersfoort, The Netherlands). Both bLF and GOS have an FDA-approved GRAS status. From week 6–9, subjects received bLF + GOS + vitamin D. Vitamin D capsules (Supra D Forte Supradyn, Berlin, Germany) contained 20 μg cholecalciferol (= 800 IE) per capsule. Maltodextrin was used as placebo product, as well as carrier for the nutritional intervention products. As placebo for vitamin D capsules, empty capsules were purchased and filled with ~250 mg maize based maltodextrin (Glucidex, IT19 premium, Roquette, Nord-Pas-de-Calais, France). bLF and GOS were mixed in bulk amount, with maltodextrin as carrier. The exact content of each component in the final powder to allow proper dosing was checked and confirmed. Powders were analyzed for potential microbiological contamination (i.e., *Bacillus cereus*, Enterobacteriaceae, yeast and fungi, total plate count, coliforms, and *E. coli*). All values were below detection, confirming the microbiological food safety of the powder batches.

Verum (“intervention”) and placebo powders were aliquoted in blinded and coded jars in a food-grade environment, in adequate amounts to allow dosing over at least 21 days. After careful instruction, participants dosed the powders at home. One dose consisted of a smoothly streaked plastic scoop that was provided, which was dissolved in water and consumed after the evening meal. Vitamin D or placebo capsules were provided in separate blinded and coded jars, and were swallowed together with the dissolved powder.

Compliance to the intake of study product was monitored by weighing of powders and counting of capsules at start and end of each intervention period. Subjects also recorded the intake of study product daily in a diary.

### Rand-36 questionnaire

At the beginning and end of the intervention study participants filled in the RAND 36-item short form survey instrument (RAND-36) questionnaire (32). The RAND-36 questionnaire is a validated questionnaire for the perception of health and well-being. The RAND-36 was analyzed according to the protocol on the subjects physical functioning, limitations in physical health, pain and general health (https://www.rand.org/health/surveys_tools/mos/36-item-short-form/scoring.html). Statistics was done by rank-transforming the data or logit transformation (only general health). A generalized linear model repeated measures ANOVA was performed.

### Registration of adverse events

Any adverse events (AE) were registered at each visit, with subjects being asked for any health complaints since the last visit. Furthermore, participants recorded AEs in a diary. The AEs were evaluated by the study physician, and judged as being or not being related to the study product (i.e., dietary intervention) or to the study procedures (i.e., blood sampling).

### Blood sampling

Blood was collected at study days 0, 21, 42, and 63 for serum storage (10 mL tubes; cat.no. 367895, BD) or for PBMC isolation (K2-EDTA; 4 × 10mL; cat.no. 367525, BD). Serum tubes were left at room temperature for at least 30 min before centrifugation at 2000 × g 10 min at room temperature. Serum was aliquoted and stored at -80°C. PBMCs were isolated within 6 h using 50 ml Leucosep tubes (227290, Greiner Bio-One) filled with Ficoll plaque plus (17-1440-02, GE Healthcare Life Sciences) according to manufacturer's protocol.

### TLR expression in pDCs and mDCs

Isolated PBMCs were stained with a TLR antibody panel (Table 2) to measure the expression of TLR2, 4, 7, and 9 *ex vivo*. To measure expression of TLRs, 2 × 10^6^ freshly isolated PBMCs/donor were stained in a 96 well plate (NUNC PP Sigma-Aldrich 7116). Per well 200 μl FACS buffer (PBS (Lonza BE17-516Q/12) + 2mM EDTA (Merck CBI 108418); 0.5% BSA (Roche 10735086001); 0.01% NaN3 (Merck CBI 822335) was added to wash the cells. Washing was done by centrifuging the plate at 400 × g for 3 min at 4°C. First, extracellular surface markers (Table 2) including 5 μl Fc block (564220, BD Pharmingen) were stained for 30 min on ice covered in aluminum foil and washed twice with cold PBS. Cells were stained with Fixable Viability Dye FVD520 (65086718, Ebioscience) in PBS and incubated for 20 min in the fridge, followed by washing the cells FACS buffer. Afterwards cells were permeabilized by adding IC fixation buffer (00-8222-49, Ebioscience) to each well and incubated for 30 min at room temperature, followed by washing twice in Perm buffer (00-8333-56, Ebioscience). The intracellular antibody mix (Table 2) in Perm buffer was incubated for 20 min in the fridge, followed by washing the cells twice in Perm buffer. Cells were resuspended in 300 ul FACS buffer and measured for 240 s on the FACS CANTO II at medium flow rate, threshold 45,000. For the panels fluorescent minus one (FMO) controls and isotype controls were included.

**Table 2:** Antibodies used for TLR expression and intracellular cytokine measurements.

**Antibody**	**Fluorochrome**	**Host**	**Isotype**	**Light chain**	**Company**	**Catalog number**	**Panel**	**Extra/intracellular**
Lineage 2	FITC	mouse	IgG1	κ	BD	643397	TLR and cytokine	Extra
HLA-DR	APC-Cy7	mouse	IgG2b	κ	Ebioscience	47-9956-42	TLR and cytokine	Extra
CD123	PE-Cy5	mouse	IgG1	κ	Ebioscience	15-1239-42	TLR and cytokine	Extra
CD11c	PE-Cy7	mouse	IgG1	κ	Ebioscience	25-0116-42	TLR and cytokine	Extra
TLR2	biotin	mouse	IgG2a	κ	Ebioscience	13992282	TLR	Extra
TLR2 ic	biotin	mouse	IgG2a	κ	Ebioscience	13472785	TLR	Extra
streptavidin	BV510				BD	563261	TLR	Extra
TLR4	BV421	mouse	IgG1	κ	BD	564401	TLR	Extra
TLR4 ic	BV421	mouse	IgG1	κ	BD	562438	TLR	Extra
FVD 520	efluor520				Ebioscience	65-0867-18	TLR and cytokine	Extra
TLR7 lc	PE	mouse	IgG2a		R&D Systems	IC5875P	TLR	Intra
TLR7 ic	PE	mouse	IgG2a		R&D Systems	IC003P	TLR	Intra
TLR9	APC	rat	IgG2a	κ	Ebioscience	17909982	TLR	Intra
TLR9 ic	APC	rat	IgG2a	κ	Ebioscience	17-4321-81	TLR	Intra
CD16	BV510	mouse	IgG1	κ	BD	740203	Cytokine	Extra
IL-6	PE	rat	IgG1	κ	Ebioscience	12706982	Cytokine	Intra
IL-6 ic	PE	rat	IgG1	κ	Ebioscience	12430183	cytokine	Intra
IFN-alpha	V450	mouse	IgG1	κ	BD	561382	Cytokine	Intra
IFN-alpha ic	V450	mouse	IgG1	κ	BD Horizon	561504	cytokine	Intra
TNF-alpha	APC	mouse	IgG1	κ	Ebioscience	17734982	Cytokine	Intra
TNF-alpha ic	APC	mouse	IgG1	κ	Ebioscience	17-4714-41	Cytokine	Intra

Flow cytometry data analysis was performed by using FlowJo software (version 10 TreeStar, Inc.) and gating was performed as is shown in Figure S1, in line with Panda et al. (8) PDCs were gated as Lineage2^−^HLA-DR^+^CD11c^−^CD123^+^ and mDCs were gated as Lineage2^−^HLA-DR^+^CD11c^+^CD123^−^. Data were exported as median fluorescent intensity for either all pDCs or mDCs per TLR.

### Intracellular cytokine measurement in pDCs and mDCs

In order to measure intracellular cytokines, 2 x10^6^ PBMCs were stimulated in a 12-well plate (CLS3513-50ea, Sigma-Aldrich) (total volume 1 ml) for 3 h in the absence or presence of PAM3CSK4 (Pam) 10 μg/ml (L2000, EMC microcollections), Ultra-pure LPS 0.1 μg/ml (3pelps, Invivogen), R848 3 μg/ml (TLRL-R848-5, Invivogen) or CpG 3 μg/ml (TLRL-2216-1 (class “A”), Invivogen) in the presence of Brefeldin A (B7651, Sigma-Aldrich) in RPMI-1640 with 5% human AB serum (H4522, Sigma Aldrich). Afterwards, cells were harvested by pipetting and stained with Lineage2, HLA-DR, CD11c, CD123, IFN-α, TNF-α, and IL-6, as described for the TLR staining. Cells were resuspended in 250 ul FACS buffer and measured for 200 s with FACS Canto II. Flow cytometry data analysis was performed by using FlowJo software (version 10 TreeStar, Inc.) and gating was performed as is shown in Figure S1. Data were exported as % cytokine-positive pDCs or mDC as % of all pDCs or mDCs.

### Cytokine and pro-inflammatory marker measurements in serum

In serum, IL-1β (558279, BD Pharmingen); TNF-α (560112, BD Pharmingen), IL-6 (558276, BD Pharmingen), sCD106 (sVCAM-1; 560427, BD Pharmingen), sCD54 (ICAM-1; 560269, BD Pharmingen), and IL-10 (558274, BD Pharmingen) were measured by cytometric bead array, according to manufacturer's protocol. Beads were measured for 50 s at high speed using a FACS CANTO II. Furthermore, IL-1RA (CHC1183, Thermo Fisher) and cartilage oligomeric matrix protein (COMP) (DY3134, R&D systems) were measured by ELISA according to manufacturer's protocol. CRP was measured with a immunoturbidemetric assay using the c802 module of Cobas 8000 from Roche. 25-OH-vitamine D was measured using chemiluminescent immunoassay using Liaison XL from Diasorin.

### Statistical analysis

Statistical analysis was performed by using IBM SPSS Statistics version 23. Data were tested for normal distribution using Shapiro-Wilk test. The intracellular cytokine production (% of all pDCs or all mDC) of pDC and mDCs per stimulation (RPMI, Pam, LPS, R848, and CpG) were analyzed by repeated measures MANOVA (RM-MANOVA). To obtain normally distributed data, percentages were logit-transformed or rank-transformed. For TLR expression the median MFI of TLR 2, 4, 7, and 9 expression was analyzed per cell type (pDC or mDC). The median MFI was 10 log transformed to obtain normally distributed data. TLR expression was analyzed by RM-ANOVA per TLR per cell type (pDC or mDC). Serum concentrations of pro-inflammatory markers (pg/ml) in serum were 10 log transformed to obtain normally distributed data. As the value 0 cannot be 10 log transformed, this value was artificially put on 0.001 to obtain a value after transformation. No RM-MANOVA could be performed on pro-inflammatory markers in serum as too many donors would be excluded, therefore an RM-ANOVA per marker was performed. After data transformation, outliers (>2SD) were removed. The transformed data were used for analysis. RM-MANOVA and RM-ANOVA were performed with additional analysis of contrasts (difference and repeated) of time^*^treatment. All statistical results are therefore a difference over time between the nutritional intervention group and placebo group. Statistically significant results are indicated in the figures as ^*^ (*p* < 0.05) or ^**^ (*p* < 0.01). As this was a nutrition intervention study with a relatively low number of individuals (*n* = 15 per group), we were also interested in statistical trends and not only in statistically significant differences. We considered trends relevant if 0.05 ≤ *p* ≤ 0.10 and indicated this in figures with a # symbol.

## Results

### Safety and tolerability

Based on the number of participants needed for the study and the inclusion and exclusion criteria, 30 elderly women were selected to participate in the study. In both the intervention group (*n* = 15) and placebo group (*n* = 15) no subjects dropped out during the study period. The intervention with bLF, GOS, and vitamin D was generally well-tolerated and safe, as only a few mild study-related AE were reported (e.g., flatulence and change in bowel habit), mainly in the intervention group. No moderately severe AE related to the study were reported. One non-study related serious adverse event was reported in the placebo group.

### Percentages of pDCs and mDCs

To determine if the nutritional intervention affected the numbers of circulating pDCs and mDCs, unstimulated PBMCs were stained with Lineage 2, HLA-DR, CD11c, and CD123. Using the gating strategy as depicted in Figure S1, the percentage of pDCs and mDCs were determined at each time point. Table 3 shows that, although there were some fluctuations in pDC and mDC percentages over time, no significant changes between the nutritional intervention and placebo group were found.

**Table 3:** The percentage of pDCs and mDC (%pDC or %mDC of all DCs) in unstimulated PBMCs (median + range) for the nutritional intervention and placebo group at study days 0, 21, 42, and 63.

	**Treatment**	**Day 0**	**Day 21**	**Day 42**	**Day 63**
%pDCs	Intervention	11.4% (3.1–45.0%)	12.6% (4.5–40.5%)	14.4% (6.0–34.2%)	13.3% (4.8–30.3%)
	Placebo	14.3% (3.3–27.7%)	13.7% (5.5–31.4%)	13.5% (4.9–31.0%)	15.9% (4.8–22.6%)
%mDCs	Intervention	68.2% (26.9–86.0%)	76.8% (50.3–87.7%)	73.2% (55.4–88.3%)	74.1% (36.1–85.8%)
	Placebo	67.6% (28.0–88.9%)	76.2% (58.9–89.8%)	77.2% (49.5–90.0%)	72.9% (37.7–86.9%)

### The effect of nutritional intervention on intracellular cytokine production in pDCs upon TLR stimulation

**Figure 2:**
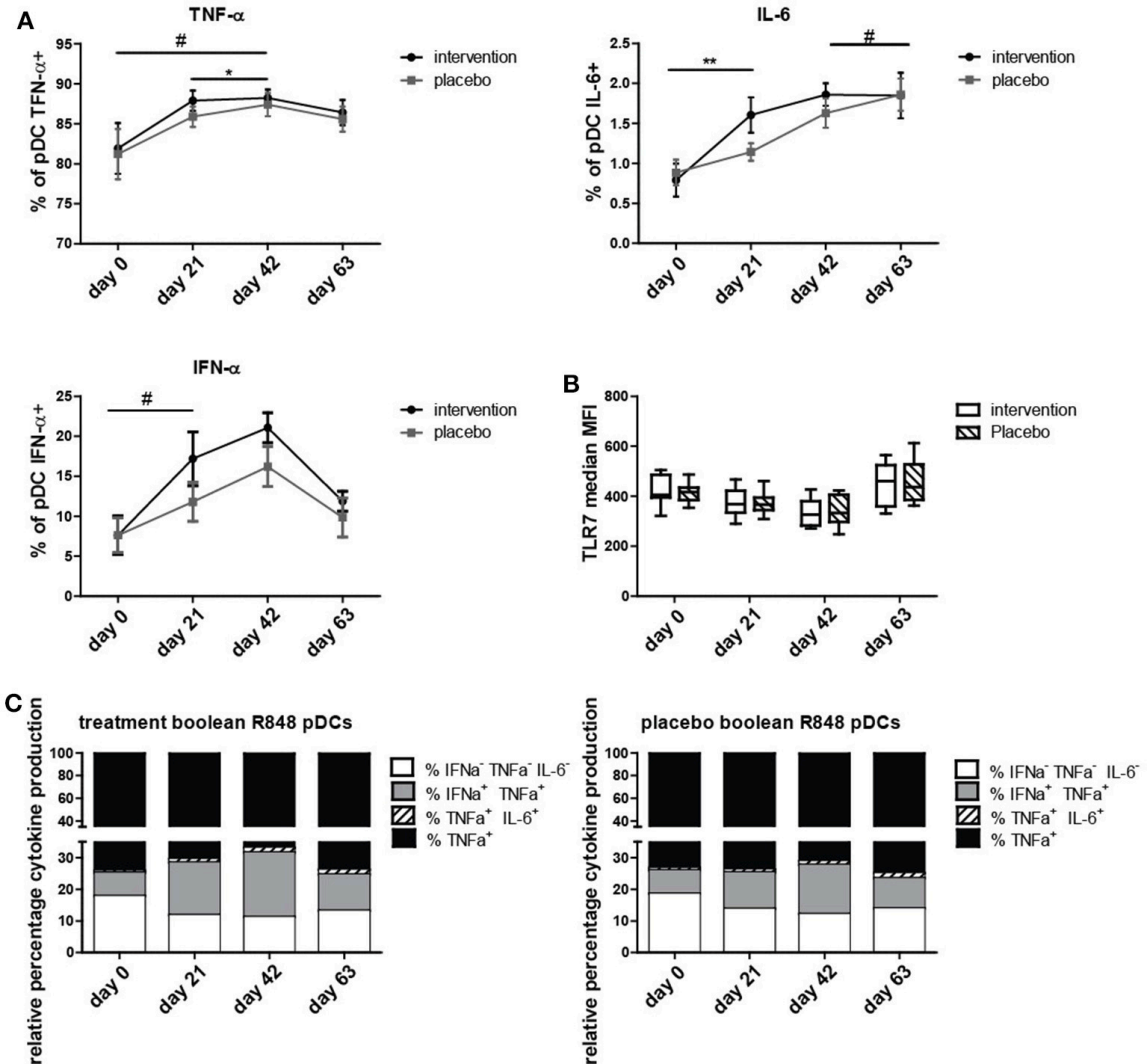
Intracellular cytokine production in pDCs upon R848 stimulation. **(A)** TNF-α, IL-6, and IFN-α positive pDCs (% positive for cytokine of all pDCs) at study days 0, 21, 42, and 63, shown as mean +/− SEM. **(B)** TLR7 expression on pDCs at study days 0, 21, 42, and 63. **(C)** Boolean gating of produced cytokine combinations only by pDCs over time. Statistical analysis was done on ranks based on logit transformed data using repeated measures MANOVA. Outliers (>2SD) were removed. ^*^p < 0.05; ^**^p < 0.01; # is given for trends, all values indicate a difference between Intervention and placebo group over time. # for IFN-α^+^ pDCs is obtained by analyzing days 0 and 21 only.

To determine if the nutritional intervention impacted the antiviral response in pDCs, intracellular IFN-α, IL-6, and TNF-α production was measured after stimulation with TLR7/8 ligand R848. The percentage of IL-6^+^ pDCs increased significantly (*p* = 0.005) and IFNα^+^ pDCs tended to increase (*p* = 0.09) at day 21 compared to day 0 in the nutritional intervention group compared to the placebo group (Figure 2). As the nutritional intervention group only consumed bLF during the first 21 days, this indicates that lactoferrin supplementation increased the response of pDCs to TLR7/8 stimulation. TNF-α^+^ pDCs tended to increase at day 42 compared to day 0 in the nutritional intervention group after TLR7/8 stimulation, while the placebo group increased from days 21 to 42.

At day 63, the number of TNF-α^+^, IL-6^+^, and IFN-α^+^ pDCs was the same in the placebo group and the nutritional intervention group after TLR7/8 stimulation. Overall, it can be concluded that bLF specifically increased IL-6 and tended to increase IFN-α production in pDCs (days 0 to 21) upon stimulation with R848, but that subsequent supplementation of GOS and vitamin D did not further enhance this effect compared to the placebo group. This increased cytokine production was not caused by an increase of TLR7 expression in pDCs of the intervention group compared to the placebo group (Figure 2B).

In order to determine which subset of cytokine producing pDCs was responsible for the increase of IL-6^+^ and IFN-α^+^ pDCs, a boolean gating strategy was performed, resulting in all eight combinations of IL-6, IFN-α, and TNF-α production from single to triple positive pDCs. Figure 2C depicts the four combinations of cytokines that were observed to be produced by pDCs. Figure 2C shows that the increase in IFN-α production is mainly due to IFN-α^+^TNF-α^+^ pDCs, and for IL-6 the majority of positive pDCs are IL-6^+^TNF-α^+^. This is in contrast to TNF-α, where the majority of TNF-α production is derived from single TNF-α^+^ pDCs.

In addition to stimulation through TLR7/8, stimulations of pDCs were performed through TLR1/2 (Pam), TLR4 (LPS), and TLR9 (CpG). In Table S1, the percentages of pDCs containing intracellular IL-6, TNF-α, and IFN-α after stimulation with Pam, LPS, and CpG are shown.

In all stimulations, a higher percentage of pDCs was positive for TNF-α compared to IL-6 and IFN-α. Even though the percentage of positive cells was low, a significant increase of IL-6 (*p* = 0.021) was observed in the nutritional intervention group compared to the placebo group after Pam stimulation at day 21, after 3 weeks of bLF supplementation. At day 21, no effects of the nutritional intervention were observed when pDCs were stimulated with LPS and CpG. At other time-points, no effects of intervention were detected in pDC, with the exception of the percentage of TNF-α^+^ pDC in response to CpG stimulation, that tended to increase in the intervention group, both from days 0 to 42 (*p* = 0.088), as well as, from days 21 to 42 (*p* = 0.085) (data not shown). Overall, these results show that enhanced cytokine production upon TLR7/8 and to a lesser extent TLR1/2 stimulation mainly occurs after 21 days in pDCs and is thus primarily the result of ingestion of bLF.

### Intracellular cytokine production in mDCs upon TLR stimulation

Next, the effect of the nutritional intervention on mDC activation was studied. For all TLR stimulations, a higher percentage of mDCs produced TNF-α, compared to IL-6 and IFN-α, see Table S2. Upon TLR1/2 stimulation, the percentage of TNF-α^+^ mDCs from the nutritional intervention-treated subjects increased compared to the placebo treated subjects at day 63 compared to day 0 (*p* = 0.03), due to a lower percentage of TNF-α^+^ mDCs at *t* = 0.

Likewise, TLR9 stimulation with CpG induced more IFN-α^+^ mDCs in the nutritional intervention group only when comparing days 63 to 0 (*p* = 0.029). At the same time, in the nutritional intervention group the percentage IL-6^+^ mDCs after TLR9 stimulation at day 63 tended to be increased in the nutritional intervention group compared to the placebo group, as IL-6^+^ mDCs in the placobo group decreases more over time. It should be mentioned however, that the IFN-α^+^ and IL-6^+^ mDC percentages after TLR9 stimulation are very low, and may not be clinically relevant.

In contrast to the results obtained in pDCs, no differences between nutritional intervention and placebo group were observed when comparing cytokine production between days 0 and 21 (bLF treatment alone), but rather from days 0 to 63 (after the complete intervention period).

### No effect of nutritional intervention on TLR expression in pDCs and mDCs

In order to analyze whether the changes in intracellular cytokine production upon TLR stimulation was due to modulation of expression of any of the TLRs, the TLR expression levels on pDCs and mDCs were measured in unstimulated PBMCs *ex vivo*. No significant differences in expression of TLR2, 4, 7, and 9 were observed for pDCs and mDCs when comparing the nutritional intervention with the placebo group over time (Figure S2), although the expression levels varied over time in both groups. This suggests that the changes in intracellular cytokines upon TLR stimulation or the differences between the intervention and placebo group were not due to differences in TLR expression levels.

### Serum markers involved in inflammation

**Figure 3:**
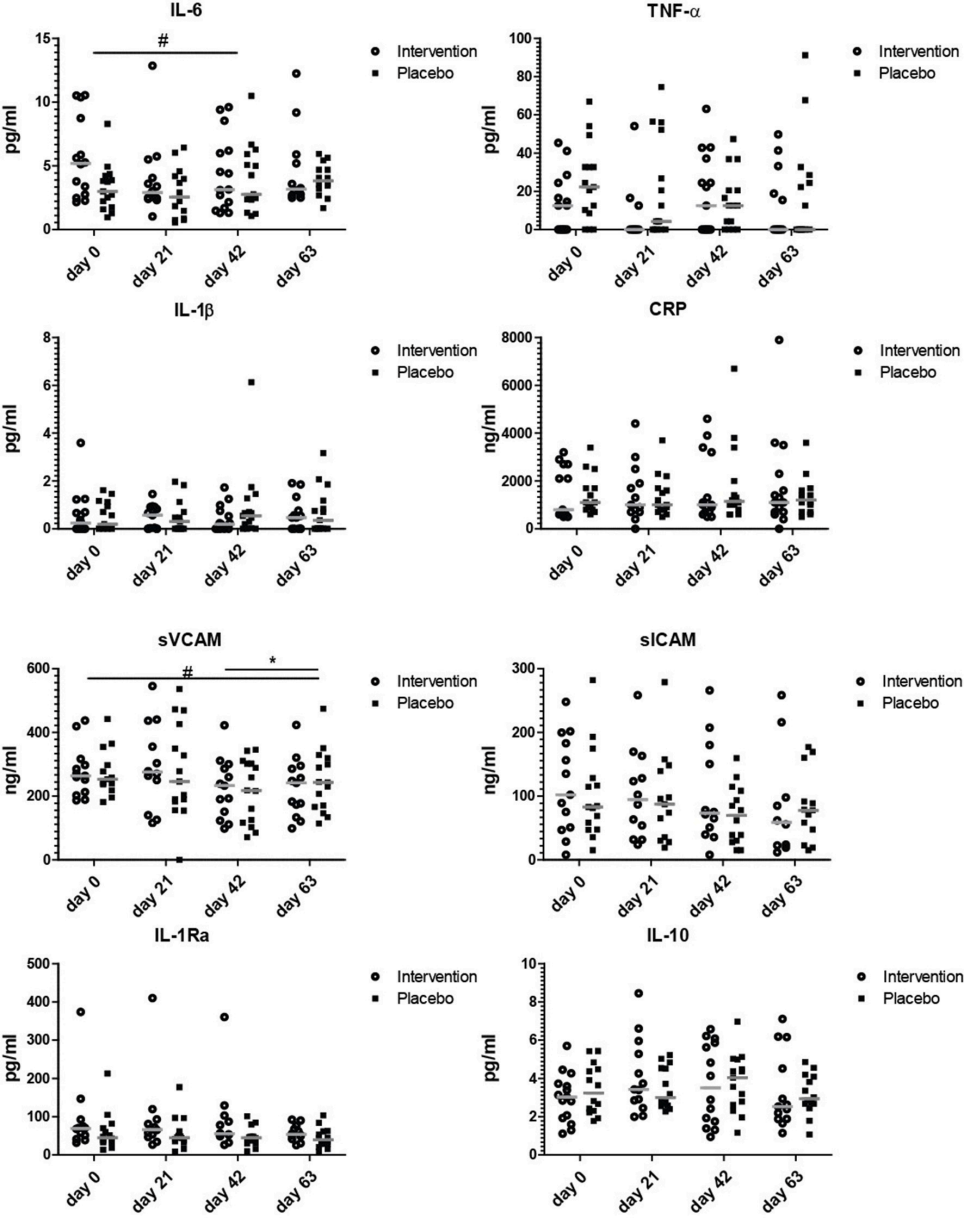
Serum markers involved in inflammation. Concentrations of IL-6, TNF-α, IL-1β, CRP, sVCAM, sICAM, IL-1RA, and IL-10 at study days 0, 21, 42, and 63. Data shown as scatter plots with median value. Analysis was done on the 10 log transformated data or ranks (IL-1β and TNF-α) by repeated measures ANOVA,. Outliers (>2 SD) were removed. All statistical differences are differences over time between the nutritional intervention group and the placebo group. ^*^p < 0.05; or # is given for trends.

To explore if the nutritional intervention affected serum concentrations of markers involved in inflammation, a wide range of aging-associated inflammatory markers was measured. Figure 3 shows that the only inflammation related marker that tended to decrease after the entire invention period was soluble VCAM, which decreased at day 63 compared to day 0 (*p* = 0.07). As there is no clear decrease of sVCAM levels from days 42 to 63 in the intervention group, the observed decrease is most likely due to a combined effect of bLF, GOS, and vitamin D. IL-6, TNF-α, sICAM, IL-1β, CRP, IL-1Ra, and IL-10 did not change during the study. Cartilage oligomeric matrix protein (COMP) as potential prognostic marker for osteoarthritis (33) was also measured in serum, as were vitamin D levels (Figure S3). Vitamin D levels did not change during the study. Although the concentration of soluble ICAM and COMP seemed to decrease gradually over time in the nutritional intervention group, this was not significant at any of the timepoints.

None of the markers was significantly changed at day 21, indicating that 3 weeks of bLF supplementation alone did not have clear effects on serum markers involved in inflammation. The only tendency to an effect on inflammatory markers was observed at day 63 after the addition of GOS and vitamin D to bLF supplementation (sVCAM).

### RAND-36 questionnaire

Table 4 shows the outcomes of analysis of the RAND-36 questionnaire in the nutritional intervention and placebo group. The questionnaire was only completed before and after the study, and thus compares health status at day 0 vs. day 63. A score of 100 is considered very healthy, a score of 0 very unhealthy. Physical function tended to improve in the nutritional intervention group (*p* = 0.09), but no effects were observed on pain, general health and limiting physical health. The results showed no major change when individuals with osteoarthritis were excluded from this analysis, indicating that the main effect of supplementation accounted for the whole group and not specifically for individuals with osteoarthritis (not shown).

**Table 4:** Mean scores and ranks for placebo and nutritional intervention group based on the RAND-36 questionaire.

	**Timepoint**	**Placebo**		**Nutritional intervention**		***P*-values (all individuals including OA)**	***P*-values (healthy individuals without OA)**
		**Mean**	**Rank**	**Mean**	**Rank**	**Time x treatment *n* = 30**	**Time x treatment *n* = 24**
Phys Func	Day 0	95	18.1	85	12.9	0.092	0.087
	Day 63	92	15.5	89	15.5		
Lim. Phys Health	Day 0	95	16	90	15	0.27	0.27
	Day 63	85	14.5	97	16.5		
Pain	Day 0	93	16.7	87	14.3	0.41	0.35
	Day 63	88	15.3	86	15.7		
General Health	Day 0	77	14.9	77	16.1	0.95	0.72
	Day 63	77	15.1	76	15.9		

*Phys Func, physical functioning; Lim. Phys health, role limitations due to physical health problems*.

## Discussion

This study demonstrates that 3 weeks of bLF supplementation increased intracellular cytokine production in pDCs in response to TLR7/8 and TLR1/2 activation. Increased responses to TLR activation were only seen in mDC after completion of the intervention study at day 63. The intervention did not show clear changes in inflammatory markers, as only sVCAM tended to decrease in the intervention group.

The rationale to study postmenopausal women aged >65 was based on several observations. TLR7 is X-linked (31) and women are found to have higher IFN-α production by pDCs compared to men (34). Furthermore, TLR7 function is reduced in postmenopausal women compared pre-menopausal women and can be improved by hormone replacement therapy, indicating that the reduced TLR7 responsiveness in elderly women can be restored (12).

pDCs are the primary producers of IFN-α upon influenza infection, which is TLR7-mediated (14). As bLF is linked to reduced number of infections by rhinoviruses and hepatitis C that are also recognized by TLR7 (19–21), we hypothesized that nutritional intervention with bLF might increase the production of the antiviral cytokine IFN-α by pDCs. Indeed, bLF supplementation alone (days 0 to 21) enhanced the production of IL-6 and tended to increase the production of IFN-α in response to TLR7/8 stimulation in pDCs. Subsequent supplementation of GOS and vitamin D did not further enhance cytokine production of pDCs in response to any TLR stimuli used compared to placebo. In contrast, supplementation of bLF during the first 3 weeks did not result in enhanced responses of mDCs to the same stimuli, nor affected serum markers involved in inflammation.

In many parameters measured in this study, the placebo group shows similar kinetics as the intervention group, which seems a time-related effect and may result in underestimation of the effects of the nutritional intervention. We hypothesized that these time-related effects were due to sunlight induced vitamin D. However, vitamin D did not significantly change in the placebo or the nutritional intervention group during the study (Figure S3). Hence, it remains elusive which time-related factor contributed to this effect in all subjects during the study period, and it stresses the need of taking along a placebo group.

In elderly, the expression of TLR1 and TLR7 on mDCs and TLR7 on pDCs was reported to be reduced compared to young adults, while the expression of TLR2 on mDCs and TLR9 on pDCs is unchanged (16, 8). Besides, a change in signaling events downstream of TLR activation occurs upon aging that leads to reduced cytokine secretion upon stimulation (4, 7). The defective TLR function in aging is illustrated by decreased cytokine production of monocytes and pDCs, as well as, mDCs of elderly people in response to ligation of TLR1/2 (35), TLR4 (36), TLR7, and TLR9 (8). In our study, the observed changes in cytokine production by pDCs after bLF supplementation were not due to increased TLR expression levels, suggesting that bLF supplementation may have improved downstream signaling of TLRs. bLF is 69% homologous to human lactoferrin on the protein level (37) and can be taken up by human cells via lactoferrin-receptor (intelectin) (38). We hypothesize that bLF exerts its effect by binding to intelectin or one of the other bLF receptors that are expressed by immune cells. These receptors are low-density lipoprotein receptor-related protein-1 (LRP-1 or CD91) (39, 40), CD14 on monocytes in complex with LPS (41), TLR4 (42), and CXCR4 (40). It is currently not known whether bLF is taken up and ends up in the blood as the whole protein or that bLF is partially digested and active peptides end up in blood, which subsequently exert an effect on pDCs.

GOS has been shown to exert an effect on inflammatory serum markers via the increase of *Bifidobacteria* levels, which are important for the production of short chain fatty acids (SCFAs), and to reduce pro-inflammatory cytokine concentrations in serum (29). However, we did not observe a reduction in pro-inflammatory cytokines in this study. This might be because our intervention period was shorter, or because the concentration of GOS was lower compared to other studies (29, 30). Another, explanation is that our group size was too small to detect significant changes in these markers, see below.

Serum concentrations of vitamin D correlate with increased IL-1β, TNF-α, and IL-6 production by monocytes upon TLR7 stimulation, while TLR7 expression is inversely correlated with vitamin D levels in serum (27). In this study, we did not observe any increase of TNF-α, IL-6, or IFN-α in stimulated pDC or mDC or changes in TLR7 expression when comparing days 42 with 63. A possible explanation is that 3 weeks of vitamin D supplementation were not sufficient to significantly enhance 25-OH vitamin D concentration in serum (Figure S3).

Interestingly, two recent publications have also reported effects of dietary intervention in elderly people on the innate immune response after TLR activation (43, 44). In the first study, a mediterranean diet did not have an effect on circulating mDC and pDC numbers, but reduced the cytokine responses of PBMC in response to TLR-mediated stimulation (44). In the second study, a mediterranean diet with additional vitamin D3 supplementation was shown to have gender-specific effects on TLR-mediated activation of PBMC in women, that in analogy with the data presented here for pDC showed increased induction of CD40^+^CD86^+^ cells upon stimulation (43).

Recent hypotheses have suggested that the decreased response to stimuli through TLR in elderly people and the enhanced steady state production of cytokines by blood cells of elderly people may have the same underlying cause, being miRNAs that regulate activation of myeloid cells downstream of TLRs (45).

It should be mentioned that the group size in this study was based on power calculations related to the primary and secondary outcomes of the study (IFN-α, TNF-α, and IL-6 production by pDCs upon *ex vivo* stimulation). For statistically significant effects on inflammatory serum markers, larger group sizes are needed (26). In addition, the population of elderly women in this study was in general healthy and mobile, with only a few subjects with Osteoarthritis (OA) (6/30). Besides, the average age was relatively low compared to other studies (46, 47). Inclusion of larger study groups including less mobile elderly women or more (non-hospitalized) women with chronic inflammatory diseases might be recommended in future studies to demonstrate effects on inflammatory markers with these ingredients.

In addition to the immune parameters described, the RAND-36 questionnaire was used to assess the health status of the subjects. The RAND-36 questionnaire results showed a trend toward improved physical function in the intervention group, as compared to the placebo. bLF has been shown to prevent arthritis in experimental animal models (48). COMP is a physiological parameter that is predictive for development of osteoarthritis (33). The tendency toward improved physical function together with the non-significant decrease in COMP concentrations in the intervention group, might suggest that nutritional supplementation with bLF, possibly in combination with GOS and vitamin D, could be relevant for prevention of osteoarthritis (OA). For practical reasons it was not possible to investigate the effect of bLF, GOS, and vitamin D in a parallel study setup with all interventions separately and combined in four active groups and one placebo group. Therefore, a longitudinal set-up with a staged introduction of bLF, GOS, and Vitamin D was chosen. However, in this study setup it is only possible to assess the effect of bLF after 3 weeks, and of the combined intervention of bLF, GOS, and vitamin D after completion of the study. As a result, we cannot conclude if the effect on physical function is the result of bLF for a prolonged period or of the GOS and vitamin D that was introduced later. However, larger studies with separate study groups including higher numbers of elderly people with OA will be needed to investigate this nutritional intervention effect further.

In conclusion, nutritional supplementation with bLF, GOS, and vitamin D is safe and enhances responses to TLR stimuli in elderly women in both pDCs and mDCs, while no clear effects on pro-inflammatory markers in serum were observed, possibly due to the study group size. Especially TLR7-mediated responses in pDCs were enhanced after bLF supplementation compared to placebo, suggesting that bLF may contribute to protection against viral infections in elderly women. The outcomes of this nutritional intervention study warrants future studies, that should be powered on the basis of the outcomes described here to confirm and extend our findings on DCs and inflammatory markers in elderly women.

## Ethics statement

This study was carried out in accordance with the recommendations of Medical Ethics Committee of Wageningen University, the Netherlands with written informed consent from all subjects. All subjects gave written informed consent in accordance with the Declaration of Helsinki. The protocol was approved by the Medical Ethics Committee of Wageningen University, the Netherlands.

## Author contributions

Study design RvN, EvH, HS, SB, and MvS. EF-V, EvH, MvS, BM, HF-B, OP, and AF execution and logistics of the study. MvS, BM, and AF data analysis. MvS, OP, SB, HS, EvH, and RvN manuscript writing and editing.

### Conflict of interest statement

MvS, OP, EF-V, EvH received research funding from FrieslandCampina to execute the intervention study. RvN is an employee of FrieslandCampina. The remaining authors declare that the research was conducted in the absence of any commercial or financial relationships that could be construed as a potential conflict of interest.

## Abbreviations

bLF: bovine Lactoferrin
COMP: cartilage oligomeric matrix protein (COMP)
GOS: Galacto-oligosacharides
mDC: myeloid dendritic cell
OA: osteoarthritis
Pam, PAM3CSK4 or (S)-[2: 3-*Bis*(palmitoyloxy)-(2-RS)-propyl]-N-palmitoyl-(R)-Cys-(S)-Ser-(S)-Lys_4_-OH
pDC: plasmacytoid dendritic cell
RM-(M)ANOVA: repeated measures (M)ANOVA
TLR: Toll-like receptors.
