# Supplementary material for: Bovine Lactoferrin Enhances TLR7-Mediated Responses in Plasmacytoid Dendritic Cells in Elderly Women: Results From a Nutritional Intervention Study With Bovine Lactoferrin, GOS and Vitamin D
Source: Front Immunol. 2018 Nov 20;9:2677. doi: 10.3389/fimmu.2018.02677 (PMC6255898; doi:10.3389/fimmu.2018.02677)
Supplement: Supplementary file 1 [file Data_Sheet_1.pdf]

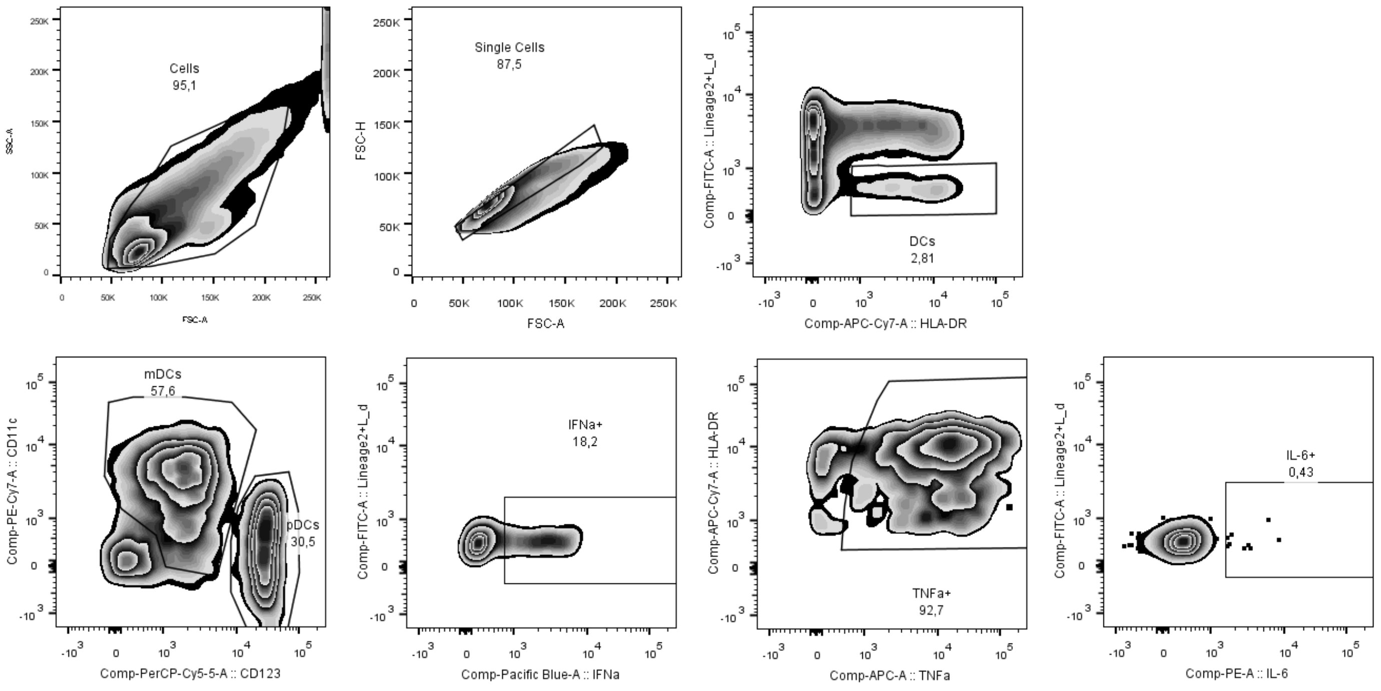

Supplementary Figure S1: Intracellular cytokine production of pDCs by a donor from the elderly population (171/002) upon TLR 7/8 stimulation with R848.

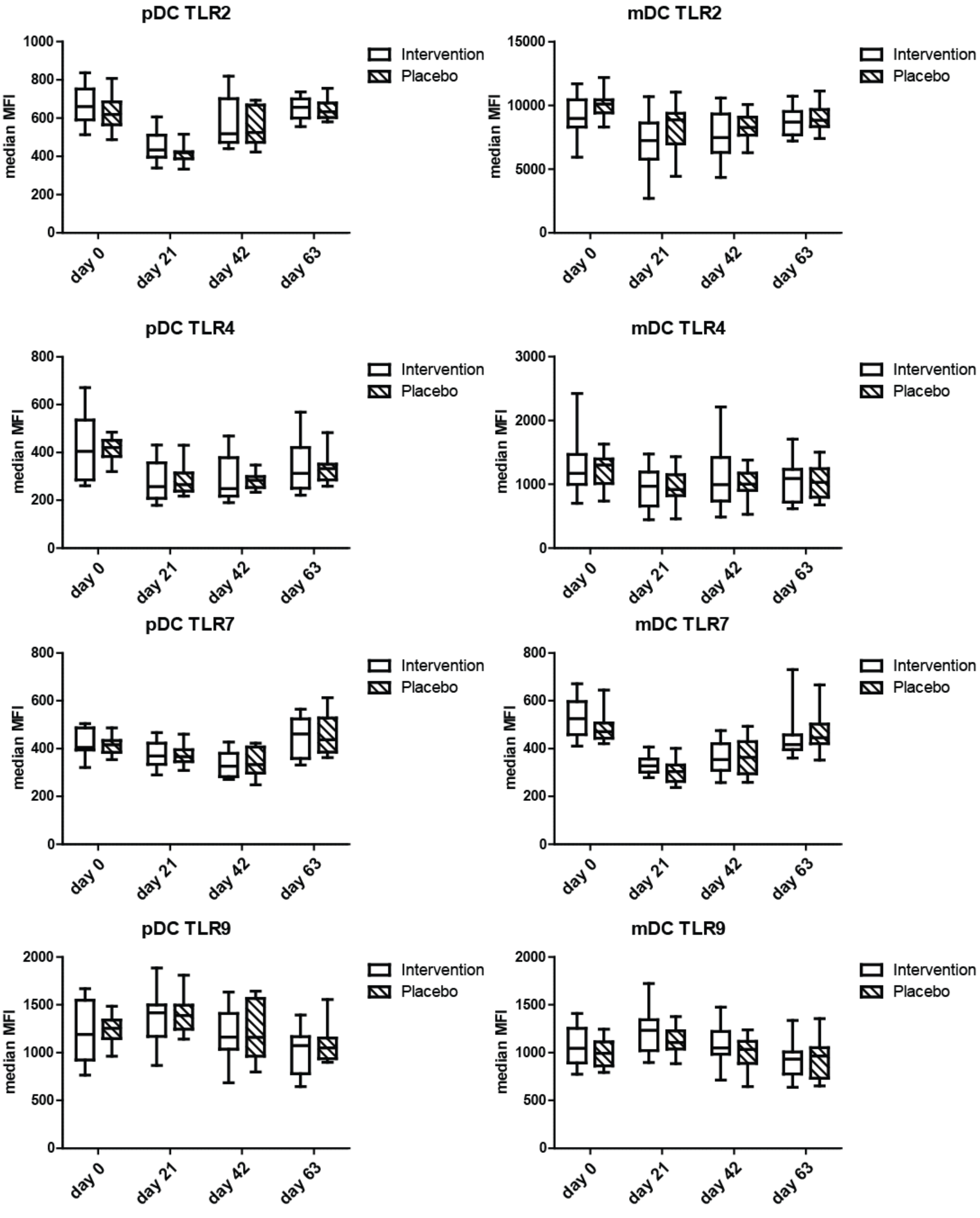

Supplementary Figure S2: TLR 2, 4, 7 and 9 expression ex vivo on pDCs and mDC at day 0, 21, 42 and 63 of the study. No significant differences were found.

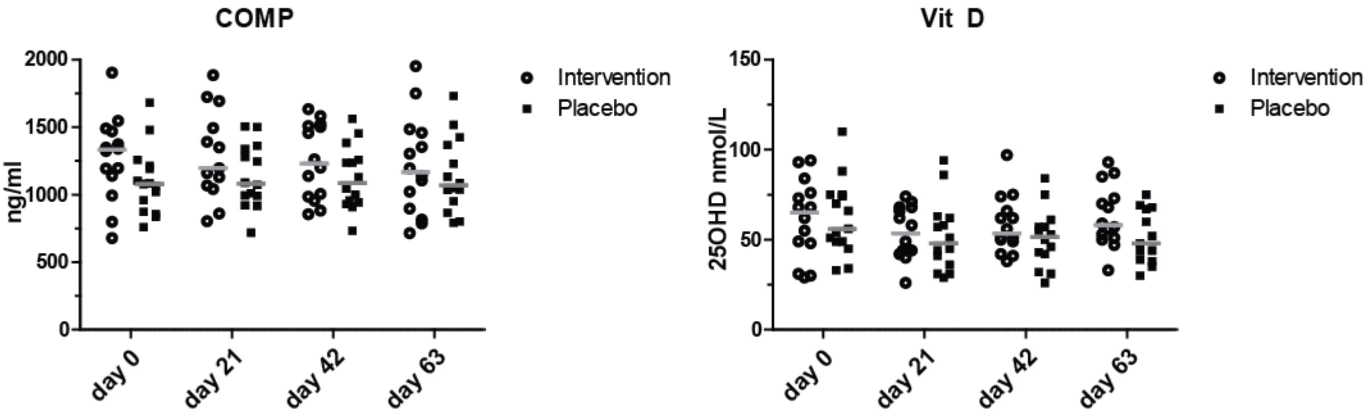

Supplementary Figure S3: Serum concentrations of Cartilage oligomeric matrix protein (COMP) and Vitamin D (25-OH-vitamin D) at study day 0, 21, 42, 63. Data shown as scatter plots with median value. Analysis was done on 10log transformed data by repeated measures ANOVA. Outliers (>2SD) were removed. No statistical differences found.

Supplementary Table S1: Intracellular cytokine production pDCs upon stimulation with Pam, LPS, R848 and CpG at day 0, 21, 42, 63. Data shown as median % + range, no outliers were removed. Intervention statically(*) increased ↑ or decreased ↓ compared to placebo over time or a trend was observed (#).

stimulation	cytokine	Treatment	Day 0 (%)	Day 21 (%)	Day 42 (%)	Day 63 (%)	Day 0 vs day 21 (p-value)	Day 0 vs day 63 (p-value)
Pam	TNF	Intervention	12.5 (3.2-19.72)	17.35 (4.04-30.62)	18.86 (3.35-31.31)	15.89 (4.27-27.16)		
		Placebo	8.16 (3.11-23.90)	6.88 (2.34-27.27)	6.37 (3.73-29.75)	7.25 (3.86-27.96)		
	IL-6	Intervention	0.2 (0.0-2.55)	0.64 (0.08-1.19)	0.68 (0.23-2.81)	0.93 (0.24-2.18)	0.021↑ (*)	
		Placebo	0.91 (0.0-1.75)	0.34 (0.1-3.38)	0.65 (0.12-2.64)	0.97 (0.1-4.66)		
	IFNa	Intervention	0.0 (0.0-0.42)	0.0 (0.0-0.05)	0.05 (0.0-0.13)	0.05 (0.0-0.11)		
		Placebo	0.08 (0.0-0.24)	0.0 (0.0-0.06)	0.01 (0.0-0.3)	0.01 (0.0-0.15)		
LPS	TNF	Intervention	1.59 (0.54-4.76)	1.7 (0.07-6.31)	1.39 (0.12-4.82)	1.44 (0.25-4.83)		
		Placebo	1.4 (0.0-8.29)	1.3 (0.21-17.28)	0.75 (0.18-18.06)	1.77 (0.12-6.02)		
	IL-6	Intervention	0.07 (0.00-2.96)	0.35 (0.0-1.18)	0.3 (0.05-1.8)	0.41 (0.06-2.68)		
		Placebo	0.24 (0.00-4.22)	0.1 (0.03-6.41)	0.16 (0.0-4.8)	0.3 (0.04-2.6)		
	IFNa	Intervention	0.00 (0.0-0.08)	0.0 (0.0-0.05)	0.0 (0.0-0.06)	0.0 (0.0-0.08)		
		Placebo	0.03 (0.0-0.13)	0.0 (0.0-0.07)	0.0 (0.0-0.06)	0.0 (0.0-0.62)		
R848	TNF	Intervention	84.62 (54.49-98.49)	88.24 (77.13-94.92)	89.08 (81.01-95.8)	86.47 (73.4-95.48)		
		Placebo	86.44 (59.34-94.58)	87.08 (76.84-94.0)	88.99 (76.02-93.32)	85.65 (73.29-94.53)		
	IL-6	Intervention	0.58 (0.0-2.26)	1.62 (0.39-3.42)	1.68 (1.04-2.92)	1.56 (1.01-5.44)	0.005↑ (*)	
		Placebo	0.89 (0.0-2.08)	1.1 (0.44-1.86)	1.64 (0.47-3.17)	1.7 (0.79-3.25)		
	IFNa	Intervention	5.0 (0.19-34.29)	12.46 (1.14-49.27)	21.87 (10.3-32.76)	13.36 (0.31-17.02)	0.09↑ (#)	
		Placebo	5.68 (0.04-28.79)	10.78 (0.61-37.75)	14.83(4.73-37.89)	6.68 (0.88-33.29)		
CpG	TNF	Intervention	0.87 (0.13-12.87)	1.55 (0.26-13.2)	2.99 (0.27-7.21)	1.85 (0.12-15.90)		
		Placebo	1.29 (0.16-18.46)	1.34 (0.18-8.87)	2.45 (0.4-5.33)	1.06 (0.19-7.1)		
	IL-6	Intervention	0.0 (0.0-0.21)	0.02 (0.00.24)	0.07 (0.0-0.23)	0.07 (0.0-0.19)		
		Placebo	0.03 (0.0-0.37)	0.03 (0.0-0.14)	0.04 (0.0-0.18)	0.06 (0.0-0.76)		
	IFNa	Intervention	0.0 (0.0-0.26)	0.0 (0.0-0.04)	0.05 (0.0-0.95)	0.0 (0.0-0.69)		
		Placebo	0.0 (0.0-0.46)	0.0 (0.0-0.6)	0.0 (0.0-0.36)	0.03 (0.0-0.15)		

Supplementary Table S2: Intracellular cytokine production mDCs upon stimulation with Pam, LPS, R848 and CpG at day 0, 21, 42, 63. Data shown as median % + range, no outliers were removed. Intervention statically (*) increased ↑ or decreased ↓ compared to placebo over time or a trend was observed (#).

Stimulation	cytokine	Treatment	Day 0 (%)	Day 21 (%)	Day 42 (%)	Day 63 (%)	Day 0 vs day 21 (p-value)	Day 0 vs day 63 (p-value)
Pam	TNF	Intervention	18.8 (0.77-41.8)	15.95 (9.96-42.7)	16.5 (9.65-42.7)	24.0 (10.2-43.1)		0.03 ↑ (*)
		Placebo	28.2 (0.66-44.5)	16.6 (9.76-35.1)	15.7 (5.31-30.3)	29.0 (10.3-36.2)		
	IL-6	Intervention	1.21 (0.08-5.28)	3.77 (1.43-4.91)	3.65 (1.77-5.59)	2.61 (1.35-3.9)		
		Placebo	1.59 (0.05-5.49)	4.54 (1.32-10.1)	3.77 (2.01-7.24)	3.33 (1.75-4.89)		
	IFNα	Intervention	0.06 (0.0-1.24)	0.02 (0.0-0.21)	0.02 (0.05-0.71)	0.08 (0.02-0.32)		
		Placebo	0.05 (0.0-0.99)	0.05 (0.01-0.22)	0.2 (0.05-0.37)	0.05 (0.02-0.1)		
LPS	TNF	Intervention	54.71 (7.1-79.82)	74.51 (58.92-85.76)	75.31 (55.64-84.86)	76.72 (56.64-86.41)		
		Placebo	65.02 (11.27-78.22)	77.52 (60.41-85.23)	77.46 (60.22-86.28)	76.46 (57.8-82.75)		
	IL-6	Intervention	33.75 (1.88-50.33)	32.83 (15.15-47.41)	36.18 (21.72-50.74)	40.06 (26.03-53.18)		
		Placebo	36.94 (0.6-51.51)	32.21 (24.68-44.81)	39.1 (18.84-48.08)	43.74 (28.85-54.02)		
	IFNα	Intervention	0.03 (0.01-0.06)	0.03 (0.0-0.11)	0.09 (0.04-0.28)	0.08 (0.01-0.16)		
		Placebo	0.02 (0.0-0.13)	0.04 (0.01-0.09)	0.11 (0.02-0.18)	0.05 (0.0-0.12)		
R848	TNF	Intervention	81.29 (53.72-96.19)	89.47 (77.92-95.69)	90.75 (79.07-95.85)	86.67 (71.72-94.31)		
		Placebo	86.75 (60.47-94.44)	91.82 (81.12-93.33)	92.47 (81.65-96.01)	87.47 (76.1-93.8)		
	IL-6	Intervention	63.47 (5.3-79.31)	46.24 (26.46-65.58)	49.99 (33.96-64.48)	55.16 (32.66-69.66)		
		Placebo	72.24 (2.33-84.3)	42.58 (31.93-50.87)	49.1 (25.79-63.71)	54.26 (33.01-66.04)		
	IFNα	Intervention	0.03 (0.0-0.12)	0.02 (0.0-0.12)	0.10 (0.00-0.29)	0.06 (0.01-0.18)		
		Placebo	0.02 (0.0-0.08)	0.03 (0.0-0.14)	0.08 (0.02-0.15)	0.04 (0.0-0.09)		
CpG	TNF	Intervention	2.9 (0.36-8.22)	2.45 (0.9-13.09)	3.28 (0.5-9.05)	5.13 (0.84-16.07)		
		Placebo	2.68 (0.21-18.09)	2.35 (0.52-14.1)	3.25 (0.47-7.92)	3.77 (0.4-17.37)		
	IL-6	Intervention	0.65 (0.33-9.47)	0.60 (0.16-1.99)	0.85 (0.29-4.31)	0.73 (0.25-1.5)		
		Placebo	0.88 (0.13-8.37)	0.77 (0.07-6.12)	0.87 (0.14-4.12)	0.59 (0.16-2.93)		0.098↓ (#)
	IFNα	Intervention	0.02 (0.00-0.21)	0.04 (0.0-0.13)	0.10 (0.03-0.22)	0.08 (0.04-0.19)		0.029↑ (*)
		Placebo	0.05 (0.00-0.20)	0.05 (0.00-0.13)	0.16 (0.05-0.26)	0.07 (0.02-0.11)		
